# Alcohol-induced liver injury in signalling pathways and curcumin's therapeutic potential

**DOI:** 10.1016/j.toxrep.2023.10.005

**Published:** 2023-10-12

**Authors:** Vetriselvan Subramaniyan, Natasha Sura Anak Lubau, Nobendu Mukerjee, Vinoth Kumarasamy

**Affiliations:** a Jeffrey Cheah School of Medicine and Health Sciences, Monash University, Jalan Lagoon Selatan, Bandar Sunway, 47500 Subang Jaya, Selangor, Malaysia; b Center for Transdisciplinary Research, Department of Pharmacology, Saveetha Dental College, Saveetha Institute of Medical and Technical Sciences, Saveetha University, Chennai, Tamil Nadu 600077, India; c Department of Microbiology, Ramakrishna Mission Vivekananda Centenary Collage, Kolkata, West Bengal 700118, India; d Department of Health Sciences, Novel Global Community and Educational Foundation, Australia; e Department of Parasitology and Medical Entomology, Faculty of Medicine, Universiti Kebangsaan Malaysia, Jalan Yaacob Latif, 56000 Cheras, Kuala Lumpur, Malaysia

**Keywords:** Alcohol consumption, Liver injury, Toll-like receptor 4 (TLR4)/NF-κB p65 pathway, CYP2E1/ROS/Nrf2 pathway, Curcumin

## Abstract

Confronting the profound public health concern of alcohol-induced liver damage calls for inventive therapeutic measures. The social, economic, and clinical ramifications are extensive and demand a comprehensive understanding. This thorough examination uncovers the complex relationship between alcohol intake and liver damage, with a special emphasis on the pivotal roles of the Toll-like receptor 4 (TLR4)/NF-κB p65 and CYP2E1/ROS/Nrf2 signalling networks. Different alcohol consumption patterns, determined by a myriad of factors, have significant implications for liver health, leading to a spectrum of adverse effects. The TLR4/NF-κB p65 pathway, a principal regulator of inflammation and immune responses, significantly contributes to various disease states when its balance is disrupted. Notably, the TLR4/MD-2-TNF-α pathway has been linked to non-alcohol related liver disease, while NF-κB activation is associated with alcohol-induced liver disease (ALD). The p65 subunit of NF-κB, primarily responsible for the release of inflammatory cytokines, hastens the progression of ALD. Breakthrough insights suggest that curcumin, a robust antioxidant and anti-inflammatory compound sourced from turmeric, effectively disrupts the TLR4/NF-κB p65 pathway. This heralds a new approach to managing alcohol-induced liver damage. Initial clinical trials support curcumin's therapeutic potential, highlighting its ability to substantially reduce liver enzyme levels. The narrative surrounding alcohol-related liver injury is gradually becoming more intricate, intertwining complex signalling networks such as TLR4/NF-κB p65 and CYP2E1/ROS/Nrf2. The protective role of curcumin against alcohol-related liver damage marks the dawn of new treatment possibilities. However, the full realisation of this promising therapeutic potential necessitates rigorous future research to definitively understand these complex mechanisms and establish curcumin's effectiveness and safety in managing alcohol-related liver disorders.

## Highlights

• Efforts are crucial in combatting alcohol's impact on society, finance, and health, with special attention to liver health.
• Unraveling the alcohol-liver link, focusing on TLR4/NF-κB p65 and CYP2E1/ROS/Nrf2 pathways, aims to clarify the intricate interaction dynamics.
• Alcohol-related liver harm requires tailored strategies to manage diverse drinking patterns and mitigate associated risks.
• TLR4/NF-κB p65 pathway's role in inflammation and immunity makes it a promising target for alcohol-induced liver injury treatment.
• Curcumin from turmeric exhibits hepatoprotective effects via TLR4/NF-κB p65 pathway inhibition, suggesting its therapeutic potential for liver ailments.
• In-depth analysis of TLR4/NF-κB p65 and CYP2E1/ROS/Nrf2 pathways expands insights, guiding precise therapies for alcohol-induced liver injury.

## Graphical Abstract

**Figure:**
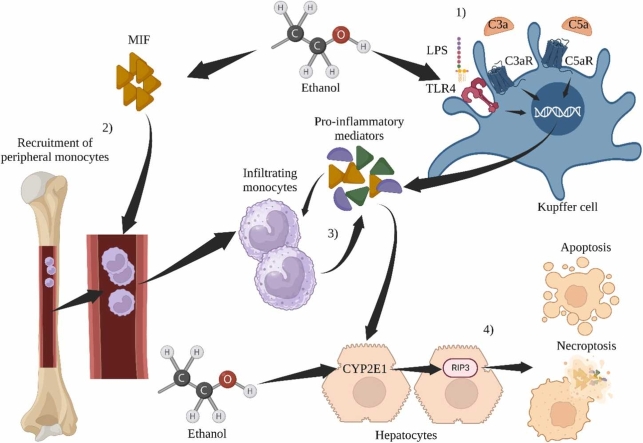

## 1 Introduction

Chronic or heavy alcohol usage constitutes a formidable hurdle, yielding profound societal, economic, and healthcare consequences, with liver damage standing out as a primary concern. Alcohol consumption varies significantly among individuals, spanning from acute, short-term to long-term, or chronic usage. This variability is shaped by a host of factors, including socioeconomic status, cultural norms, religious practices, gender, age, and personal health conditions [1], [2]. The 2016 Global Alcohol per Capita Consumption (APC) report revealed that high-income nations and specific regions within Africa, Asia, and Latin America recorded higher APC rates. Conversely, Middle Eastern countries and nations with predominantly Muslim populations reported lower rates [3]. Overconsumption of alcohol is associated with a broad range of health issues, including both communicable and noncommunicable diseases and targeted organ dysfunction. In 2016, alcohol-related disorders made a significant impact on global disability-adjusted life years (DALYs), with liver cirrhosis, especially in the Western world, emerging as a severe consequence of heavy alcohol usage. Remarkably, the highest DALYs due to liver cirrhosis were observed in India, while countries like Brunei Darussalam, Iceland, Kuwait, Qatar, and Oman reported the lowest [4], [5], [6].

In Malaysia, a discernible upsurge in chronic hepatitis B (CHB) and chronic hepatitis C (CHC) incidence has been noted, with particular ethnic groups presenting higher prevalence rates of certain liver diseases. Given that the liver is integral to alcohol metabolism, accounting for nearly 98% of alcohol breakdown, it is uniquely susceptible to harm. This damage can spiral into severe conditions such as cirrhosis, cancer, and even higher mortality rates. The liver is central to various physiological functions, including metabolism, secretion, storage, and detoxification. Therefore, dysfunction in this organ can precipitate a variety of degenerative, inflammatory, and non-inflammatory diseases. These disorders can be triggered by a myriad of factors, including infections, autoimmune disorders, exposure to toxins, and excessive alcohol consumption [7], [8].

**Fig. 1:**
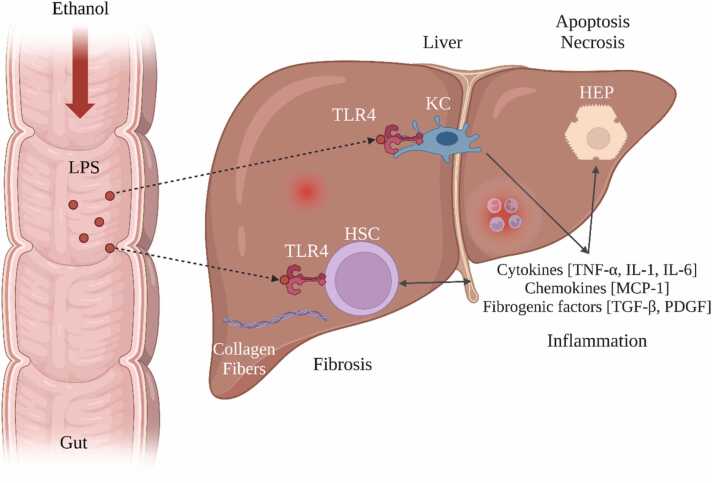
Illustrates the relationship between alcohol-induced liver disease (ALD) and the liver-gut microbiota axis.

Alcohol-associated liver disease (ALD), encompassing fatty liver, hepatitis, and cirrhosis, is a significant fallout of excessive alcohol intake and is a leading cause of chronic liver damage and mortality worldwide. Fig. 1 exhibits that the pathogenesis of ALD involves complex processes such as protein-aldehyde adduct formation, immune reactions, lipid peroxidation, and cytokine release [9], [10], [11], [12], [13], [14]. The liver-gut microbiota axis, influenced by genetic, dietary, and environmental factors, holds a crucial role in preserving liver health. Perturbations in the intestinal barrier can adversely affect liver health, with alcohol-induced alterations in gut microbiota leading to an increase in pro-inflammatory mediators in the liver, thereby altering the gut microbiome composition. Alcohol's direct impact on liver cells can result in abnormal intestinal barrier functions, changes in gut microbiota, and activation of toll-like receptors in liver cells. These series of events significantly contribute to the development of liver diseases [15], [16]. Moreover, the activation of reactive oxygen species (ROS), interleukin-6 (IL-6), and interleukin-8 (IL-8) in the liver can add to liver toxicity through the overexpression of TNF-α mRNA [17].

Excessive alcohol consumption and the subsequent metabolic fallout contribute to a rise in the production of reactive oxygen species (ROS), which is a key contributor to liver damage. This harmful effect largely originates from a reduced antioxidant response within the liver [18]. Alcohol-associated liver disease (ALD) is a wide-ranging disorder, encompassing conditions from simple steatosis or fatty liver to more severe liver damages like cirrhosis, steatohepatitis, and hepatocellular carcinoma [19]. The detrimental effects of alcohol on various tissues and organs occur mainly due to acetaldehyde generation, an upsurge in ROS, and activation of pro-inflammatory and other signalling pathways [20].

Pioneering research has found similarities between the effects of alcohol-induced hepatic steatosis in rat models and humans, thereby reinforcing the utility of such models for understanding the pathology of human ALD. Ethanol, a hepatotoxin, incites damage to liver cells, driven by oxidative stress and an inflammatory response, following its metabolic breakdown in the liver [21], [22]. On a metabolic level, ethanol degrades through three oxidative pathways, with the enzyme alcohol dehydrogenase (ADH) playing a crucial role in liver parenchymal cells. ADH aids in the conversion of ethanol into its metabolic intermediate, acetaldehyde, marked by a functional formyl group [23].

Our review sets out to broaden the existing understanding of alcohol-induced liver injury by examining the complex mechanisms involved, especially the oxidative stress and inflammatory response triggered by alcohol metabolism in the liver. Additionally, we delve into the potential protective role of specific compounds, like curcumin, against the negative effects of alcohol. This novel approach brings a fresh perspective to ALD research and could potentially set the stage for groundbreaking therapeutic interventions to tackle this worldwide health problem.

## 2 The global impact of alcohol intake on liver disorders

Excessive alcohol consumption poses a significant international health threat, making up about 5.1% of all disability-adjusted life years (DALYs) in 2016, equating to a massive 132.6 million DALYs globally. In Western societies, alcohol consumption is identified as the primary factor behind liver cirrhosis. High per-capita DALYs linked to alcohol-related liver cirrhosis are seen in countries such as India, the United States, China, Nigeria, and Indonesia. In contrast, the lowest rates are documented in countries like Brunei Darussalam, Iceland, Kuwait, Qatar, and Oman [2]. Liver malignancies related to alcohol intake follow similar trends, with countries such as China, Vietnam, Russia, Thailand, and India presenting the highest DALYs. Conversely, the lowest rates are reported in Brunei Darussalam, Sao Tome and Principe, Djibouti, Qatar, and Bhutan [2]. In the context of Malaysia, from 2010 to 2018, there has been a noticeable rise in chronic hepatitis B (CHB) and chronic hepatitis C (CHC) cases, primarily affecting the Chinese population for CHB and the Indian community for alcohol consumption. Interestingly, the Malays have the highest incidence of hepatitis C-related liver cirrhosis [24], [25]. These statistics underscore the substantial global health impact of liver diseases related to alcohol consumption.

The liver, which metabolizes about 98% of ingested alcohol, is highly susceptible to potential alcohol-induced injuries, including cirrhosis and carcinogenesis. Data from Australia indicates that approximately 2% of alcohol consumers develop alcohol-induced cirrhosis each year, with a disturbing median survival rate of 1–2 years [4]. Considering the critical functions of the liver - metabolism, secretion, storage, and detoxification - the implications of liver disorders are far-reaching, extending to degenerative conditions (cirrhosis), inflammatory diseases (hepatitis), and non-inflammatory conditions (hepatosis) [26].

Excessive alcohol intake leads to ALD, a leading cause of chronic liver damage and mortality worldwide. The progression of ALD, which includes fatty liver, alcohol-related hepatitis, and cirrhosis, is chiefly instigated by the hepatotoxic properties of alcohol, which sets off a series of adverse metabolic responses. The formerly held belief that malnutrition is the main driver behind ALD has been replaced by the understanding that alcohol metabolism is the primary trigger of a complex pathological sequence involving protein-aldehyde adduct formation, immune activation, lipid peroxidation, and cytokine release [27], [28], [29]. Emerging evidence underscores the significance of the liver-gut axis, emphasizing its substantial interaction with the gut microbiota. Alcohol-induced disruptions in gut microbiota can lead to intestinal barrier dysfunction, alterations in the epithelial lining and mucous membranes, and disturbances in gut-liver interactions, ultimately contributing to liver diseases [30]. The overexpression of TNF-α mRNA in liver cells and macrophages triggers a pro-inflammatory response, escalating the production of reactive oxygen species (ROS), culminating in hepatic toxicity [31], [32].

Further studies have revealed that alcohol-induced liver damage results from a combination of factors, including the generation of acetaldehyde, ROS, and the activation of pro-inflammatory and other signalling pathways [33], [34], [35]. Analogous findings between human and rat studies indicate that alcohol alone can induce hepatic steatosis. Alcohol, acting as a hepatotoxin, incites oxidative stress and a corresponding inflammatory response in the liver [36], [37], [38]. Notably, alcohol dehydrogenase (ADH) plays a crucial role in alcohol metabolism, transforming ethanol into acetaldehyde in liver parenchymal cells [39], [40]. These insights provide a foundation for future exploration aimed at understanding and potentially mitigating the global health burden of alcohol-associated liver diseases.

## 3 Unveiling the therapeutic potential of curcumin in hepatic wellness

Natural compounds often possess potent therapeutic attributes that can be harnessed to combat a myriad of health conditions, including hepatopathies [41]. The global medical fraternity is witnessing a growing interest in botanical treatments, which owe their burgeoning appeal to their easy accessibility, minimal toxicity, wide-ranging pharmacological attributes, and fewer side effects when juxtaposed with synthetic drugs [42], [43].

Curcumin, a naturally occurring polyphenol derived from the rhizome of Curcuma longa or turmeric, stands out in this domain. Owing to its favourable safety profile and potential medicinal efficacy, curcumin has emerged as a compelling subject for health science research [44]. In traditional Asian medicinal practices, curcumin has been an extensively employed therapeutic agent attributed to its multifaceted health-promoting characteristics, such as antioxidative, anti-inflammatory, antimutagenic, antimicrobial, and anticancer activities [45]. Being a polyphenol, curcumin exhibits broad biological activity, as it can interact with several cellular signalling pathways, thereby substantiating its extensive health benefits [46], [47], [48], [49], [50], [51], [52], [53], [54], [55], [56], [57], [58].

**Fig. 2:**
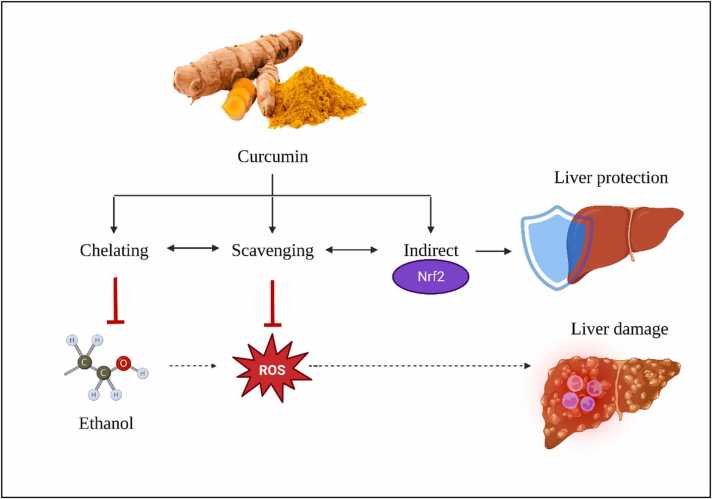
Protective effect of curcumin against ethanol-induced liver damage, highlighting the molecular mechanisms involved.

A plethora of clinical and preclinical investigations underscore the therapeutic prowess of curcumin in mitigating various human diseases. Of particular note is curcumin's hepatoprotective capacity against environmental and occupational toxic compounds. As we continue to explore the therapeutic potential and underlying molecular mechanisms of natural compounds such as curcumin, we pave the way toward more comprehensive and innovative strategies for managing liver disorders as expressed in Fig. 2.

## 4 Curcumin's multidimensional hepatic defence mechanisms: a comprehensive exploration

The bioactive compound curcumin, predominantly found in turmeric, has garnered significant attention for its intricate hepatoprotective attributes against detrimental effects posed by heavy metals such as arsenic, cadmium, and mercury. At the forefront of its hepatoprotective arsenal lies its formidable capability to combat cellular degeneration and lipid breakdown in hepatic tissues. Integral to this protective stance is its pivotal role in maintaining the equilibrium of the antioxidant glutathione (GSH), an indispensable agent in cellular resilience and homeostasis. Furthermore, curcumin's proficiency in modulating and fortifying the activity of endogenous antioxidant enzymes fortifies the liver's mitochondrial integrity [59].

On a molecular plane, curcumin's defence against heavy metal-induced hepatic distress is rooted in its unparalleled antioxidant and chelating characteristics. A crucial component of this defence mechanism is its activation of the Nrf2/Keap1/ARE signalling cascade, an axis fundamental for cellular resistance against oxidative insults. Curcumin's expansive hepatoprotective repertoire encompasses antioxidant, anti-inflammatory, anti-cholestatic, anti-fibrogenic, and anti-carcinogenic domains. By orchestrating these diverse mechanisms, curcumin adeptly manages hepatic inflammation, truncates oxidative disturbances, elevates the synthesis of detoxification enzymes, and thwarts undue hepatic stellate cell activity, all the while bolstering mitochondrial performance. Its strategic modulation of the Keap1/Nrf2/ARE pathway underpins its holistic approach to hepatoprotection [60], [61]. In light of its multifaceted hepatoprotective strategies, curcumin's potential as a cornerstone in pre-emptive and therapeutic interventions for oxidative stress-induced liver ailments is undeniable. This compound's multifunctional molecular interactions warrant exhaustive scientific investigations, particularly within the purview of hepatic disease intervention.

## 5 The dual-pronged role of toll-like receptor 4 (TLR4)/NF-κB p65 in disease progression: An analytical perspective

**Fig. 3:**
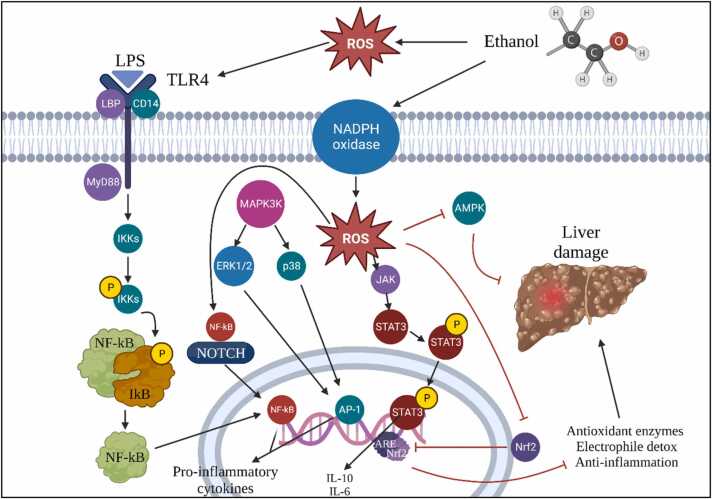
Reactive Oxygen Species (ROS) Signaling Pathways in Ethanol-Induced Cellular Responses: Implications for Inflammation and Hepatic Damage.

Historically, the Toll-like receptor 4 (TLR4) has been lauded for its adeptness at detecting exogenous molecular patterns, predominantly those originating from pathogenic invaders. This includes its recognized interaction with entities like lipopolysaccharide (LPS) associated with gram-negative bacterial species. Yet, contemporary research has expanded our comprehension of TLR4's multifaceted roles (Fig. 3). It has been unveiled that TLR4 is equipped to recognize endogenous molecules, especially those released from damaged or necrotic cells, termed damage-associated molecular patterns (DAMPs) [62], [63]. When TLR4 interfaces with DAMPs, a potent pro-inflammatory cascade is initiated, a crucial element of the innate defense framework. While inflammation inherently serves a restorative purpose, its unchecked escalation can pose dire repercussions for the organism. The ramifications of TLR4's activation extend across a vast clinical spectrum. Its involvement has been pinpointed in numerous pathological states, inclusive of microbial-induced infections and non-infectious conditions like ischemic events, neurodegenerative maladies, and an array of neurological disorders. Notably, there exist contexts where TLR4 activation can, paradoxically, amplify disease progression, underscoring its multifaceted impact on health [64], [65], [66], [67], [68], [69], [70]. Owing to TLR4's nuanced role in both pathogenic interception and tissue damage mediation, it is imperative to conduct in-depth scientific analyses. Such endeavours are vital to decipher its full therapeutic potential across a diverse range of clinical landscapes.

## 6 Deciphering the complex interplay between toll-like receptors and NF-κB in mammalian inflammation dynamics

In the mammalian immune defence arsenal, toll-like receptors (TLRs) emerge as a set of highly-conserved transmembrane proteins. Integral to the immune response mechanism, these proteins are specifically attuned to detect and respond to a plethora of pathogen-associated molecular patterns (PAMPs) from various microbial sources. Among the TLRs—ranging from TLR1 to TLR11—TLR4 has been of paramount interest. Its key involvement in directing several inflammatory signalling pathways, including the TLR4/NF-κB, TLR4/IRF3, and TLR4/PI3K/Akt pathways, underscores its importance in immune modulation [71].

Nuclear factor-κB (NF-κB) acts as a central player in various cellular processes. Its role encompasses immune responses, inflammation mediation, and cell growth and differentiation. Balanced regulation of NF-κB is imperative, as any deviation can precipitate numerous health complications. Disorders ranging from autoimmune diseases and inflammatory conditions to malignancies like rheumatoid arthritis and certain types of cancers can be traced back to its aberrant activity. As a result, inhibitory strategies targeting NF-κB signalling are becoming cornerstone approaches in therapeutic endeavours for cancer and inflammation treatment [72].

The molecular makeup of the NF-κB family is diverse, with five primary components: p65 (RelA), RelB, c-Rel, p105/p50 (NF-κB1), and p100/52 (NF-κB2). They are capable of forming both homo- and heterodimeric complexes, with the p50/65 combination being the most prevalent. However, others, like the p65/c-Rel and p65/p52 configurations, also exist and are expressed in specific cellular contexts [73]. Under basal conditions, NF-κB dimers remain inactive in the cytoplasm, held in check by the inhibitors known as IκBs (Inhibitors of κB). The role of IκBs is pivotal; by sequestering NF-κB proteins, they prevent unwanted activations. However, when cellular signalling dictates, IκBs are phosphorylated and subsequently degraded, leading to the activation and nuclear translocation of NF-κB complexes [74].

**Fig. 4:**
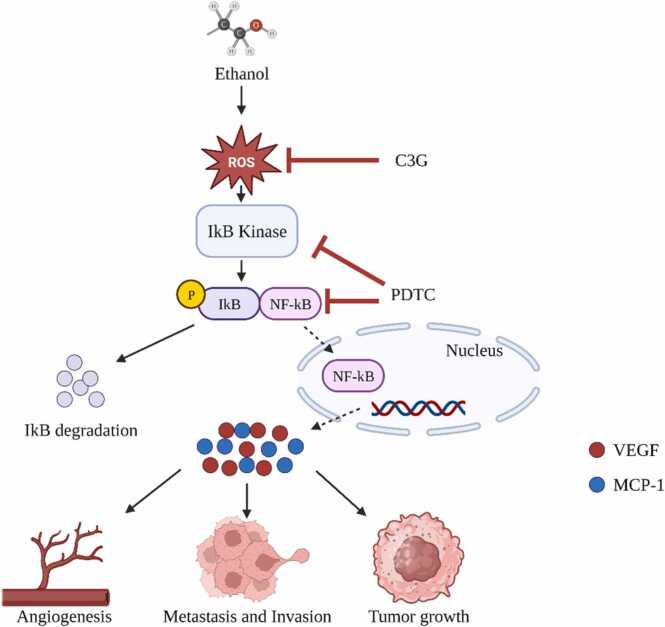
Involvement of the NF-kB Signalling Pathway in Ethanol-Enhanced Progression of Hepatocellular Carcinoma.

Fig. 4 illustrates the NF-κB system is characterized by its unique DNA-binding subunits. Activated by a myriad of external stimuli related to infection and inflammation, NF-κB's activation follows one of two paths. The first, or the canonical pathway, is defined by the degradation of IκB. The second, the non-canonical pathway, is demarcated by the processing of precursors p100 and p105. These distinct pathways underscore the intricate regulatory mechanisms that ensure a finely-tuned NF-κB response [75], [76], [77], [78], [79], [80], [81], [82], [83], [84], [85], [86], [87], [88], [89], [90], [91].

In conclusion, understanding the sophisticated relationship between the TLR4/NF-κB p65 signalling and the broader NF-κB family is crucial for developing advanced therapeutic strategies. Given their central role in inflammation and the associated pathological conditions, further research into these molecular entities promises to yield novel insights and breakthroughs in disease management.

## 7 Decoding the TLR4 and NF-κB synergy in alcohol-induced hepatic distress

Long-standing alcohol consumption undeniably casts a shadow over liver health. Central to this destructive narrative is the interplay between two molecular powerhouses: the Toll-like receptor 4 (TLR4) and the nuclear factor kappa-light-chain-enhancer of activated B cells (NF-κB). Elucidating their collaborative role in the genesis and trajectory of alcohol-associated liver disease (ALD) holds the key to understanding this pathology's essence [92]. Chronic alcohol exposure disarrays gut microbial equilibrium, resulting in heightened gut wall permeability to bacterial endotoxins, notably lipopolysaccharide (LPS). These toxins, once in circulation, rendezvous with the liver, particularly with TLR4 receptors on Kupffer cells (KCs) - the liver's sentinels [93], [94]. This interaction initiates a domino effect of inflammatory responses, implicating signalling agents like myeloid differentiation factor-2 (MD-2), eventually culminating in an endemic inflammatory milieu in the liver. Simultaneously, alcohol's prolonged exposure tweaks the otherwise protective NF-κB signalling, rendering it hyperactive. This altered state aggravates inflammation through the upregulation of pro-inflammatory genes. This heightened inflammatory state further accentuates liver injury, coupled with a surge in fibrosis—a hallmark of ALD progression [95].

**Fig. 5:**
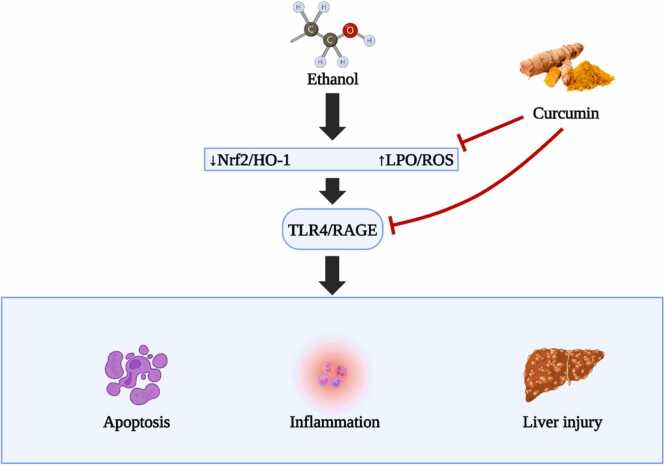
Depicts the interconnected roles of TLR4 and NF-κB signaling in the development of alcohol-induced hepatic distress.

In synthesis, the interdependent roles of TLR4 and NF-κB form the crux of alcohol-inflicted hepatic trauma. While TLR4 kindles the initial inflammatory spark, NF-κB fans the flames, driving chronic liver injury and fibrosis [93], [95]. Recognizing these pathways as potential therapeutic goldmines could usher in innovative interventions, potentially reversing the devastating impacts of alcohol on the liver. Crafting interventions to recalibrate these pathways might be the next frontier in ALD management (Fig. 5).

## 8 Unraveling the complex dynamics of NF-κB p65 activation and its implications

The p65 subunit, a vital member of the NF-κB transcription factor collective, is renowned for its dynamic activation in response to an expansive variety of stimuli. Though fleeting, this activation catalyzes the amplification of numerous target genes, orchestrating myriad cellular undertakings—ranging from cell proliferation and inflammatory mediator release to the induction of programmed cell death. The p65 activation trajectory is safeguarded by intrinsic control systems, fortifying the equilibrium within this intricate cellular nexus. An essential pivot in p65 activation is its journey into the nucleus, orchestrated by specialized proteins known as importins. Specific importins, such as importin α3, α4, α5, and β1, strategically engage with the nuclear localization signal (NLS) of p65. They operate in a sequence, either replacing or competing in a harmonized fashion, ultimately driving the p65's entry into the nucleus. Intriguingly, cellular context defines the expression dynamics of these importins, which in turn modulates the nuclear transition of p65, thereby shaping its transcriptional influence. p65's role in target gene modulation thrives amidst a complex milieu of interactions with other transcriptional factors. Some amplify its effects, like AP-1, while others, such as cEBP/β, serve as inhibitors [96]. This intricate matrix is further nuanced by post-translational modifications, including degradation, ubiquitination, acetylation, methylation, and phosphorylation, which evolve in a hierarchical manner, defining the ultimate trajectory of NF-κB p65 activation. These modifications critically sculpt various aspects of p65's functionality—ranging from its spatial distribution to interaction potential and stability. In essence, the intricacies governing p65 activation, ranging from importin dynamics to post-translational modifications, offer a profound insight into its physiological and pathological implications. Such insights are paving the way for therapeutic strategies, promising targeted interventions in inflammation-driven disorders and cancerous transformations [97].

## 9 Decoding the p65 pathway's role in alcohol-related liver disease: A deep dive into inflammatory mechanisms and therapeutic opportunities

Chronic alcohol-induced liver disease (ALD) has consistently piqued scientific interest. One crucial element under investigation is the NF-κB p65 signalling pathway's involvement in the progression of ALD. An intricate dance between the p50 and p65 NF-κB dimers offers a comprehensive understanding of liver complications arising from sustained alcohol consumption. In its basal state, these dimers are sequestered in the cytoplasm, effectively inhibited by the protein IκB. However, upon the liver's interaction with pro-inflammatory agents, namely TNF-α and IL-6, a cascade ensues. The phosphorylation and subsequent degradation of IκB facilitate the nuclear translocation of NF-κB, where it regulates genes pivotal to the inflammatory response [98].

On the metabolic front, alcohol undergoes a transformation into acetaldehyde, a deleterious metabolite. Notably, acetaldehyde is implicated in the heightened activation of IκBα kinase, pivotal for IκBα phosphorylation. This process subsequently enhances NF-κB activity, promoting the release of inflammatory mediators—central to the pathology of ALD.

Adding another layer of complexity, TNF-α, an output of NF-κB activation, reciprocally augments NF-κB p65's expression. This feedback mechanism perpetuates liver inflammation. Recent murine studies have further elucidated alcohol's interplay with the p65 pathway, underscoring the pathway's significance in alcohol-mediated liver complications. Harnessing a deeper understanding of this pathway might be the linchpin in developing interventions to counteract the deleterious impacts of chronic alcohol consumption on hepatic health [99].

## 10 Curcumin and the TLR4/NF-κB/p65 pathway: Exploring therapeutic frontiers in alcohol-associated liver ailments

Derived from turmeric, curcumin is lauded for its remarkable anti-inflammatory and antioxidant properties. Its hepatoprotective role, especially against agents like alcohol, is well-acknowledged, though the precise mechanisms remain a subject of intense research. Emerging studies have elucidated curcumin's multifaceted protective effects against alcohol-mediated hepatic complications [100].

Moreover, curcumin's anti-inflammatory potential, realized through the modulation of nuclear factor B and c-Jun N-terminal kinases, holds promise in neurodegenerative contexts, such as Alzheimer's disease. Given its therapeutic arsenal, there's an increasing consensus that curcumin might also serve as a protective agent against alcohol-induced neuronal impairments [101], [102], [103], [104], [105], [106]. Its unique ability to modulate oxidative stress and inflammation positions curcumin as a potential therapeutic linchpin in hepatorenal disorders linked to inflammation and oxidative derangements [101], [102], [103].

## 11 The CYP2E1/ROS/Nrf2 axis in alcohol-related liver pathogenesis: Bridging biochemical mechanisms to clinical insights

The central nervous system's (CNS) interplay with alcohol results in a plethora of behavioral manifestations. A key player in this nexus is acetaldehyde, a product of alcohol oxidation. The CNS concentrations of this metabolite are predominantly governed by localized ethanol metabolism, given its inability to navigate the blood-brain barrier. The central protagonists in this metabolic narrative are catalase and cytochrome P450 2E1 (CYP2E1), with the latter's role in the brain being of prime significance [104].

Recent academic discourse underscores CYP2E1's pivotal role in the CNS ethanol-to-acetaldehyde conversion. Found across various tissues and cell types, CYP2E1, especially within mitochondria, might be central to initiating oxidative stress during acetaldehyde synthesis. Moreover, the inherent properties of metallic nanoparticles underscore their potential in modulating reactive oxygen species (ROS) production, which in turn influences a gamut of cellular processes [105], [106].

**Fig. 6:**
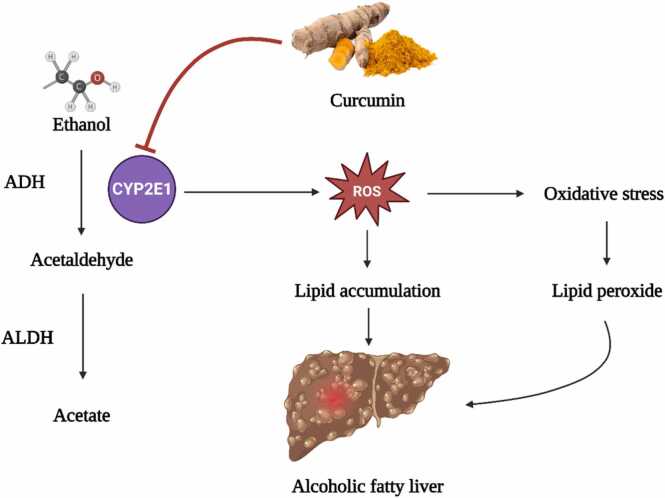
Illustrates the critical CYP2E1/ROS/Nrf2 axis and its involvement in alcohol-induced liver pathogenesis.

In summation, the interrelation between CYP2E1, ROS, and Nrf2 signalling is central to alcohol-induced liver complications [107]. Delving deeper into this triad offers a promising avenue for therapeutic innovations, addressing the detrimental outcomes of alcohol on hepatic health (Fig. 6).

## 12 Deciphering the interplay of CYP2E1, ROS, and Nrf2 in the landscape of ethanol-induced liver harm

The Nrf2 mechanism, more formally known as nuclear factor erythroid 2-related factor 2, emerges as a central regulator, crafting cellular defences against oxidative challenges. Acting as a master switch, it modulates the transcription of genes harbouring antioxidant response elements, thus determining cellular responses to oxidative stresses. Within this intricate network, CYP2E1 occupies a central stage. Notably, its adeptness at processing a gamut of harmful compounds, notably ethanol, and transforming them into potentially detrimental derivatives such as acetaldehyde and 1-hydroxyethyl radical, merits attention. Furthermore, CYP2E1 paves the way for the generation of reactive oxygen species (ROS), setting the backdrop for oxidative turmoil [108].

Innovative methodologies like the intragastric ethanol infusion technique have painted a clear linkage between elevated CYP2E1 levels, lipid peroxidation, and the progression of liver complications tied to ethanol. An intriguing avenue of research is whether curbing CYP2E1 can modulate lipid peroxidation and potentially ward off these liver complications. Such an exploration demands a deeper understanding of the multifaceted mechanisms through which CYP2E1 influences liver health [109].

Ethanol's metabolic odyssey via CYP2E1 brings us face to face with a diverse array of ROS, encompassing hydrogen peroxide (H2O2), hydroxyl radicals (OH-), and other carbon-based radicals. While our innate antioxidant mechanisms endeavour to counter these ROS, habitual alcohol consumption skews this balance, depleting pivotal reserves such as mitochondrial glutathione (GSH). This imbalance renders hepatocytes vulnerable and magnifies their sensitivity to agents like TNF-α, thereby heightening cell mortality [110].

The narrative is further complicated as ROS exhibits a propensity to activate pathways involving c-Jun N-terminal kinase (JNK) and the activator protein 1 (AP-1) transcription factor. Such activations precipitate a cascade of events, leading to cellular over-proliferation and lipid peroxidation. Even more daunting is the potential of lipid peroxidation byproducts to form DNA structures with carcinogenic potential. Evidence of such configurations has been documented in liver tissues from patients suffering from alcohol-related liver disorders and other inflammatory, oxidative stress-driven conditions [109], [111].

The microsomal ethanol oxidizing system (MEOS) introduces another layer of complexity. Here, the enzyme CYP2E1, which requires NADPH and is magnified by sustained alcohol exposure, operates. As MEOS processes ethanol to acetaldehyde, it simultaneously releases ROS. The grim twist to this tale is the bolstering of CYP2E1 by habitual alcohol consumption, leading to amplified ROS output and augmented oxidative stress. Habitual alcohol consumption weaves a narrative of liver disorders by inundating the liver with reactive agents. This deluge diminishes antioxidants, culminating in molecular aberrations via lipid peroxidation. Supercharged CYP2E1 by alcohol turns into a ROS powerhouse in the liver. However, our body's inherent defense strategies, epitomized by agents like peroxiredoxin 1 (Prx1) and sulfiredoxin 1 (Srx1), offer a formidable resistance. The presence of Prx1 in the endoplasmic reticulum, juxtaposed with CYP2E1, and the rejuvenating prowess of Srx1, become pivotal in ROS containment. Furthermore, Nrf2, awakened by sustained alcohol exposure and elevated CYP2E1 levels, steers the transcription of antioxidant enzymes. The strategic activation of Nrf2 offers a promising protective umbrella against alcohol's damaging effects on the liver. Within the realm of alcohol metabolism, the trinity of enzymes - alcohol dehydrogenase (ADH), CYP2E1, and aldehyde dehydrogenase (ALDH) - stands in the limelight. While moderate alcohol exposure seems to invigorate ADH activity, chronic exposure dampens ADH, thrusting CYP2E1 to the fore for alcohol metabolism. This shift, however, is not devoid of pitfalls, as CYP2E1 also releases deleterious byproducts. In this scenario, the natural wonder, curcumin, emerges as a potential modulator, revitalizing the muted ADH activity and tempering CYP2E1 excesses, fostering a safer metabolic trajectory for alcohol [111].

Delving into the sub-cellular impacts of alcohol, ROS emerges as a principal disruptor, especially within mitochondria. Such intrusions manifest as mitochondrial dysregulation, marked by dwindling ATP reserves, rampant lipid peroxidation, and mitochondrial DNA disruptions. Curcumin, armed with its robust antioxidant faculties, provides a protective shield, neutralizing these radicals and fortifying cellular boundaries [108].

In essence, consistent alcohol intake casts a shadow on liver health, primarily via ROS and oxidative distress. While CYP2E1's role in processing ethanol and producing ROS is undeniably pivotal, the combined might of our natural defences, supplemented by agents like Nrf2 and curcumin, illuminate pathways of hope. These synergies offer tantalizing glimpses into potential therapeutic strategies, aiming not only to prevent but perhaps even reverse the scars left by alcohol on liver landscapes [110].

## 13 Nrf2: The cellular sentinel in the face of ethanol-driven liver ailments

The molecular guardian, Nrf2, or as scientifically termed, nuclear factor erythroid-derived 2-like 2 (NFE2L2), is not just another transcription factor. It acts as the vanguard of cellular defenses, heeding distress signals, and mobilizing the regiments of antioxidant enzymes and detoxification proteins. Upon activation, Nrf2 navigates to the cell's operational hub - the nucleus. Here, it allies with antioxidant response elements (AREs), enhancing the transcriptional activity of pivotal genes and forming the bulwark against oxidative agents and electrophiles. Curcumin, derived from turmeric, has been identified as a potent Nrf2 activator. Through its stimulation of the Nrf2 pathway, curcumin augments the cellular defence repertoire, providing broad-spectrum protection across various tissues and conditions. In the context of liver challenges, especially those stemming from toxins, curcumin's defensive mechanisms appear intertwined with Nrf2 activation. By augmenting the output of antioxidant enzymes, Nrf2 mitigates ROS and curbs oxidative harm, the primary culprits underpinning ethanol-induced liver injuries [99], [110], [112].

**Fig. 7:**
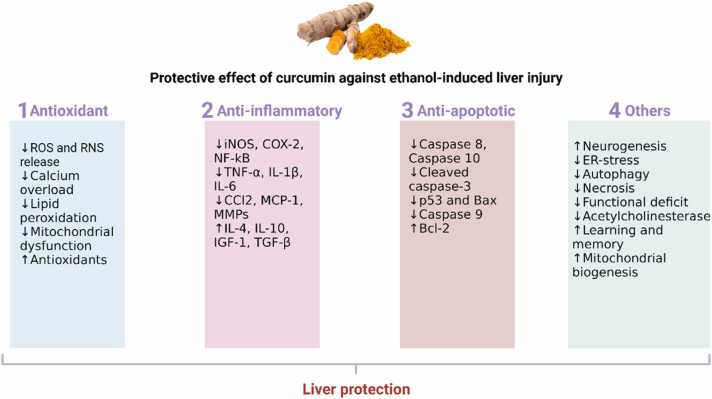
Protective mechanisms of curcumin.

The nuanced relationship between curcumin, Nrf2, and the antioxidant machinery is still an evolving story. Delving deeper into the dynamic interactions between these players could unearth novel therapeutic horizons, heralding advances in managing and potentially circumventing ethanol-induced liver challenges (Fig. 7).

## 14 Curcumin: Nature’s solution to ethanol-induced liver damage

Curcumin, the bioactive compound in turmeric, acts as a robust protector against ethanol-induced liver damage through various mechanisms. It functions as a potent antioxidant, counteracting reactive oxygen species (ROS) produced during ethanol metabolism, safeguarding liver cells. Curcumin also displays impressive anti-inflammatory capabilities, mitigating the inflammation triggered by alcohol consumption. Additionally, it regulates the enzyme CYP2E1, reducing harmful metabolite production and liver damage. Furthermore, curcumin activates the Nrf2 pathway, strengthening the liver's antioxidant defenses, and enhances antioxidant enzyme activities like SOD, CAT, and GPx. It adeptly modulates the IκBα–NF-κB pathway to reduce inflammation. Moreover, curcumin protects cells from apoptosis, supports mitochondrial health, and holds promise as a versatile therapeutic agent for liver health in the context of alcohol-related challenges [108], [105], [112].

Curcumin, through its vibrant tapestry of interactions, stands tall as a beacon of therapeutic promise. As shown in Table 1, Curcumin has diverse roles, spanning from antioxidant champion to cellular guardian, highlighting its potential to address liver challenges. As we delve deeper into this golden gem, curcumin continues to enchant, offering glimmers of hope for holistic liver health.

**Table 1:** Summary of the effects of curcumin on TLR4/NF-κB p65 and CYP2E1/ROS /Nrf2 signalling pathways.

Signalling pathways	Effects	Target study / treatment	Type of animal study	Source
TLR4	Inhibition of TLR-4 Inhibition of Downstream Signalling Pathway: NF-κB, IRF3, MyD88, and TIRF Downregulation of Proinflammatory Cytokines TNF-α, IL-1, IL-2, IL-6, IL-8, and Mitogen-Activated Protein Kinase (MAPK) Suppression of Oxidative Stress Enzymes: Nitric Oxide Synthase, Cyclooxygenase, and Lipoxygenase	Acute inflammatory injury Experimental traumatic brain injury Cerebral Ischemia-Reperfusion Necroptosis inflammation in liver	Mice Rats Mice and rats Chicken	[92] [93] [94] [95]
NF-kB	Inhibition of the NF-κB Inflammatory Signalling Pathway Inhibition of Asthma Severity via HDAC1 (H3acK9) Modulation and NF-kB Suppression in a Mouse Model of Allergic Asthma Reduction of NF-kB and TNF-α Levels Decreased Expression and Translocation of NF-κB and p-NF-κB	Traumatic spinal cord injury Airway inflammation and fibrosis Pain intensity after high-intensity physical exercise Demyelination disease as diabetes mellitus (DM)	Rats Balb/c mice Clinical study Schwann cells (SCs)	[96] [97] [98] [99]
p65	Inhibition of NF-κB-p65 Translocation Reduces the Release of Inflammatory Factors Reduced p65 phosphorylation Inhibition of Proliferation and Expression of VEGF and NF-κB p65 Mitigation of Boldenone-Induced Neurobehavioral Disturbances in Rats: Normalization of Oxidant/Antioxidant Balance and Suppression of TLR4/MyD88/TRAF-6/NF-κB Pathway and Downstream Proinflammatory Signaling Molecules TNF-α and IL-1β	Lumbar intervertebral disc degeneration Pulp inflammation HRCECs induced by high glucose Boldenone-induced aggression in rats	Rats Human dental pulp stem cells (hDPSCs) Human retinal capillary endothelial cells (HRCECs) in vitro studies Rats	[100] [101] [102] [103]
CYP2E1	Inhibition of CYP2E1 Overexpression Induced by ACR in Liver and Kidney Tissues Inhibition of Cytochrome P4502E1 (CYP2E1), Sterol Regulatory Element-Binding Protein-1c (SREBP-1c), and Acetyl-CoA Carboxylase Production Hindering Profibrogenic Transcripts Linked with Myofibroblasts and Mouse Hepatic Fibrosis Enhancement of Alcohol Dehydrogenase (ADH) and Aldehyde Dehydrogenase (ALDH) Regulation: Effects on CYP2E1 Activity, Reactive Oxygen Species (ROS), Bax, Bcl-2, and Inflammatory Mediators IL-1β, IL-6, and TNF-α	Acrylamide-induced hepatic and renal impairment Alcohol-induced Fatty Liver Disease N, N′‑methylene bis acrylamide‑induced liver damage and hepatic cancer cell growth Hepatic toxicity	Rats Mice Mice (Mus musculus) Rats	[104] [105] [106] [107]
ROS	Increased ROS Level: Regulation of Cellular Redox Status and Mitochondrial Function Promotion of Glutathione Synthesis: Aiding in Recovery from Ethanol-Induced Liver Damage Exhibiting Hepatoprotective Effect against CYP-Induced Hepatotoxicity in Rats Inhibition of High Glucose-Induced Inflammatory Injury in RPECs by Interfering with the ROS/PI3K/AKT/mTOR Signalling Pathway	Mitochondrial function Ethanol-induced oxidative stress Cypermethrin Toxicity Glucose‑induced inflammatory injury	Mice Zebrafish Rats Human retinal pigment epithelial cells (RPECs)	[108] [109] [110] [111]
NRF2	Activation of NRF2 Signalling Pathway: Cellular Protection against Oxidative Injury	Acetaminophen-induced liver injury	Rats	[112]

## 15 Unlocking curcumin's power: The ultimate shield against alcohol's assault on the liver

Recent research showcases its effectiveness in reducing indicators of liver distress, and its potent antioxidant abilities combat alcohol's oxidative aftermath. Curcumin's dosage-dependent benefits, ranging from suppressing lipid peroxidation to rejuvenating the glutathione pathway, demonstrate its adaptability. It orchestrates an intricate antioxidant ballet, amplifying crucial enzymes like SOD and GPx to maintain liver resilience. This versatile compound's promise extends to liver ailments, offering a holistic approach to rejuvenation. Despite its bioavailability challenges, innovations like nanoparticle-infused curcumin and adjuvants like piperine enhance its clinical potential. Curcumin's role as a fortress against alcohol-induced liver damage underscores its significance in liver health, awaiting further research to solidify its legacy [113], [114].

## 16 Encore: Curcumin's grand promise

To encapsulate, curcumin stands tall as a formidable fortress against the ravages of alcohol-induced liver damage. Its multi-layered roles, combined with its natural prowess, herald it as a beacon in liver health. As we stand at this exciting juncture, only in-depth research and meticulously designed clinical trials will sculpt its undeniable legacy in safeguarding liver vitality.

## 17 Discussion

The consequences of alcohol consumption on liver health have long been established, with biochemical pathways like TLR4/NF-κB p65 and CYP2E1/ROS/Nrf2 at the crux of the relationship. These pathways, when disrupted, can induce inflammatory reactions and oxidative stress, both pivotal in the onset of alcohol-associated liver disease (ALD). Our comprehensive research journey elucidates the groundbreaking role of curcumin, an antioxidant and anti-inflammatory powerhouse, in countering ethanol-induced liver damage, with a spotlight on its modulatory effects on these integral pathways [115].

By casting curcumin in the central narrative of liver health, we are ushering in a new epoch of understanding. Historically, while curcumin has been revered for its therapeutic benefits, our findings provide a fresh dimension by focusing on its adaptability and responsiveness to varying alcohol-induced stressors. This is accentuated by the nano-micelle technology, a pioneering stride that addresses curcumin's bioavailability challenge [116], [117]. Such enhancements position curcumin not merely as a beneficial compound but as a potent therapeutic agent with a magnified efficacy. From the perspective of the TLR4/NF-κB p65 pathway, curcumin’s attributes as an anti-inflammatory agent are underscored. Given the commonality of NF-κB dysregulation in ailments including ALD, curcumin's potential to intervene becomes paramount. Concurrently, its antioxidative prowess has a significant role in harmonizing the CYP2E1/ROS/Nrf2 pathway, thereby neutralizing the oxidative stress pivotal in alcohol-induced liver degeneration. Curcumin shows promise in clinical applications, but rigorous research is crucial to confirm its safety and effectiveness across various health conditions [118], [119].

The evidence underscoring curcumin's role in liver enzyme regulation further authenticates its potential benefits. Yet, optimal dosages and supplementation durations are territories yet to be fully chartered [120]. The molecular machinations guiding curcumin's hepatoprotective impact also call for deeper, more detailed inquiries. While curcumin’s potency in managing diverse chronic conditions is documented, there's a pressing need for exhaustive studies within expansive and varied patient demographics to ascertain its efficacy in ALD treatment [121].

The potential synergy between curcumin and other therapeutic agents remains an exciting frontier that could redefine ALD treatment paradigms. Furthermore, the amalgamation of breakthrough technologies, such as Proteolysis-Targeting Chimeras (PROTACs), offers an exhilarating prospect in the ALD therapeutic domain. By synergizing with the body's intrinsic protein degradation machinery, curcumin-infused PROTACs might herald a new age of therapeutic precision and efficacy [122], [123]. To encapsulate, our research posits curcumin as a beacon of hope against alcohol-induced liver damage. Yet, unlocking its full potential demands an interdisciplinary, deep-dive research approach, which will not only demystify curcumin's hepatoprotective mechanisms but also pioneer novel treatment modalities, capitalizing on innovations like PROTACs. PROTACs leverage curcumin's properties by linking it to a molecule that homes in on particular proteins for degradation inside cells, enabling the targeted breakdown of disease-related proteins [124], [125], [126]. Curcumin from turmeric exhibits potent hepatoprotective effects through diverse mechanisms. Curcumin has been reported to stimulate liver regeneration by promoting the proliferation of hepatocytes and activating growth-promoting pathways like the Wnt/β-catenin signaling pathway. This may aid in the recovery of liver function following injury [127], [128].

## 18 Conclusion and future prospects

In synthesizing the complex interactions between alcohol consumption and liver damage, this review highlights two crucial biochemical pathways: TLR4/NF-κB p65 and CYP2E1/ROS/Nrf2. The TLR4/NF-κB p65 pathway plays an integral role in inflammation and immune responses, with dysregulated NF-κB implicated in numerous pathological conditions, including alcohol-related liver disease (ALD). On the other hand, the CYP2E1/ROS/Nrf2 pathway significantly contributes to oxidative stress and antioxidant responses, which are pivotal elements in the onset and progression of ethanol-induced liver damage.

Curcumin, a phytochemical derived from turmeric, has shown remarkable promise in counteracting these deleterious effects due to its robust antioxidant and anti-inflammatory properties. The hepatoprotective capacity of curcumin is likely mediated through its inhibitory effects on the TLR4/NF-κB p65 pathway, thus offering a potential mechanistic framework for therapeutic intervention in conditions such as ALD. Our analysis further indicates that short-term curcumin supplementation could reduce liver enzyme levels, implying potential benefits for various liver diseases. However, despite these promising insights, our understanding of the underlying mechanisms of curcumin's hepatoprotective action remains incomplete. Therefore, future research efforts should prioritize unravelling these mechanisms to optimize the therapeutic application of curcumin. Furthermore, comprehensive evaluations of curcumin's safety and efficacy in larger, diverse patient populations are warranted.

Considering future prospects, the integration of curcumin into Proteolysis-Targeting Chimeras (PROTACs) technology represents a promising frontier for investigation. PROTACs are an emerging class of drugs designed to exploit the cell's natural protein degradation machinery to remove specific proteins from within the cell. They have shown potential for addressing drug resistance, enhancing the selectivity of therapeutic agents, and reducing side effects. Given the broad-spectrum biological activities and well-established safety profile of curcumin, the development of curcumin-based PROTACs may provide a novel strategy for enhancing the therapeutic efficacy and specificity of curcumin, particularly in ALD and other alcohol-related liver disorders. Furthermore, curcumin-PROTACs might be tailored to target specific proteins implicated in the TLR4/NF-κB p65 and CYP2E1/ROS/Nrf2 pathways, thereby selectively modulating these pathways and enhancing the hepatoprotective effects of curcumin. However, the successful design, synthesis, and clinical application of curcumin-PROTACs will require significant advancements in our understanding of the molecular targets of curcumin and the intricacies of protein degradation pathways.

In conclusion, while curcumin presents a compelling therapeutic option for alcohol-induced liver damage, the journey towards its clinical application will necessitate comprehensive investigations into its mechanism of action, safety, efficacy, and potential synergistic effects when combined with other treatment modalities. Incorporating emerging technologies such as PROTACs into these investigations could lead to innovative strategies for managing alcohol-related liver injuries, opening new avenues for combating this significant public health concern.

## CRediT authorship contribution statement

Conceptualization, **V.S.**, **N.S.A.L.**, **V.K**, and **N.M.**; methodology, **V.S.**; resources, **V.S.**, **N.S.A.L.**, **V.K**, and **N.M.**; writing—original draft preparation, **V.S.**, and **N.S.A.L.**; writing-review and editing, **V.S.**, and **N.S.A.L.** All authors have approved the content of the submitted manuscript.

## Declaration of Competing Interest

The authors declare that they have no known competing financial interests or personal relationships that could have appeared to influence the work reported in this paper.

## Data Availability

The authors are unable or have chosen not to specify which data has been used.
